# Catalytic pyrolysis of waste polypropylene using low-cost natural catalysts

**DOI:** 10.1038/s41598-023-37769-8

**Published:** 2023-07-20

**Authors:** A. I. Eldahshory, Karim Emara, M. S. Abd-Elhady, M. A. Ismail

**Affiliations:** 1 Department of Mechanical Engineering, Faculty of Engineering, Beni-Suef University, Beni-Suef, Egypt; 2 Department of Mechanical Engineering, Faculty of Engineering, Helwan University, Helwan, Egypt; 3 Mechatronics Department, Faculty of Engineering and Materials Science, German University in Cairo (GUC), Cairo, Egypt

**Keywords:** Fossil fuels, Renewable energy, Chemical engineering

## Abstract

The objective of this research is to produce oil from the catalytic pyrolysis of waste polypropylene (WPP) using a low-cost natural catalyst. Three natural catalysts were examined, i.e. Kaolin, Hematite, and white sand. Different catalyst-to-plastic ratios were examined, i.e. 1:1, 1:2, 1:4, 1:6, and 1:8. The utilized catalysts were elementally analyzed using the XRF analysis and the surface area was analyzed by the BET multi-point method. The WPP thermal degradation behavior was investigated by the thermogravimetric analysis (TGA), then the generated liquid oil was analyzed using the gas chromatography-mass spectrometry (GC–MS) and the differential scanning calorimetry (DSC). Thermal cracking without a catalyst produced a yield of 70 wt% of liquid oil, and the maximum oil yield in case of using Hematite and white sand as a catalysts were 70 wt% and 68 wt%, respectively. However, the ratio of 1:2 of the Kaolin to the WPP produced the highest oil yield of 80.75 wt%, and the ratio of 1:8 of the white sand to the WPP produced the highest gas yield, i.e. 44 wt%. Using Kaolin in the catalytic pyrolysis of WPP produced oil with the lowest percentage of heavy oils, i.e. 25.98%, and the highest percentage of light oils, which is 25.37%, when compared to other catalysts such as Hematite and white sand. Kaolin has the lowest cost of oil production compared to Hematite and white sand, which is 0.28 $/kg of oil. Kaolin is an economical catalyst that improves the quality, as well as the quantity of the produced oil in comparison to Hematite, white sand and the non-catalytic case.

## Introduction

Globally, continual scientific and technological progress has led to an increase in the consumption of energy. The consistent excess in energy consumption has caused fossil fuel reserves depletion and increasing the environmental damage caused by gas emissions^1, 2^. The supply of fossil fuels is expected to steadily deplete after 2042^3^. Consequently, there is a critical need to develop new and sustainable forms of energy. Various plastic products are essentially consumed items in our daily life. Egypt's growing population and the raising life requirements led to the consumption of large amounts of plastic^4^‏. Plastic materials are used in numerous applications due to their lightweight, low cost, durability, and possessing wide range of domestic and commercial usage^5^. The lack of sufficient waste management methods of disposing plastic waste has led to serious environmental problems^6^. Egypt is the Arab world's largest plastic polluter, with 5.4 million metric tons of plastic produced annually^7^. In 2019, Worldwide Life revealed that Egypt was the biggest source of Mediterranean plastic pollution, pouring about 250,000 tons a year^8^.

The typical global use of plastic is about 35% polyethylene (PE), 23% of polypropylene (PP), 13% of Polyvinyl chloride (PVC), 10% of polystyrene (PS), 7% of Polyethylene terephthalate (PET) and 12% of other polymers. The most prevalent plastics in polymer waste streams are PE and PP, followed by PS^9^. Polypropylene is a multifunctional polymer, due to its excellent mechanical characteristics, low density, and its high chemical resistance. Polypropylene is found in furniture, pipes, office folders, storage boxes, computer chips, medical bottles, generic containers, and also the auto sector^10^. Hydrocarbons are utilized in the production of oxidants, plastic stabilizers, and flame retardants. As a result, Plastics are not biodegradable and would persist in the environment for a very long time^11^. Therefore, there are vast quantities of waste plastics in landfills with no noticeable degradation^12, 13^. Subsequently, recycling plastic waste is a very important and critical issue for reducing landfill buildup. Accordingly, Plastic waste recycling into valuable products, e.g., liquid and gas fuels, by thermal cracking^14–17^, which is known as pyrolysis, is an optimal solution for waste plastic disposal. Pyrolysis is the Thermal cracking of long hydrocarbons chain into shorter-chain or smaller molecules at high temperatures of 300–800 °C without oxygen^18^. The gas produced by plastic pyrolysis has a high calorific value (HCV) because of the presence of hydrogen, ethane, methane, butane, and propane, and the produced liquids can be utilized instead of conventional fuel. Plastic waste is regarded as one of the most sustainable sources of fossil fuels due to its HCV compared to diesel and gasoline^19, 20^. Typically, catalysts are utilized in plastic pyrolysis to enhance product selectivity and distribution. Additionally, catalysts are utilized to foster the dispersion of hydrocarbons and to upgrade pyrolysis products^21^. Thus, catalysts are used to produce liquid oil with properties comparable to conventional fuels like diesel and gasoline^22^.

Plastic waste pyrolysis has been carried out during the last decade using a variety of catalysts. In plastic pyrolysis, there are three categories of catalysts: zeolite, silica-alumina, and fluid catalytic cracking (FCC)^23^. Zeolites catalysts were used by Susastriawan and Sandria^24^ to catalytic pyrolysis of polyethylene, and the production of liquid fraction was increased by decreasing zeolite size and increasing temperature. Onwudili et al.^25^ investigated catalytic pyrolysis of many polymers employing catalysts such as ZSM-5 zeolites Y, and FCC. It has been concluded that the catalyst resulted in a drop in the liquid fraction yield. Even though the quantity of aromatic compounds increased, the quality of liquid fraction improved making it viable as a fuel. Linh and Tuan^26^ used a rock-reforming metal and ZSM-5 as catalysts to get liquid products from the pyrolysis of polypropylene, and it has been concluded that the used catalyst drastically decreased the alkenes in the liquid products.

Wang et al.^27^ used HZSM-5 as a catalyst to perform catalytic rapid co-pyrolysis of polycarbonate wastes (PCW) to produce aromatic hydrocarbons. It is reported that the catalytic conversion of PCW using HZSM-5 made it easier to create aromatic hydrocarbons compared to non-catalytic pyrolysis. Akubo et al.^28^ used Y-zeolite catalysts loaded with transition metal promoters at 1% and 5% metal loadings of Ni, Fe, Ga, Mo, Co, and Ru to analyze the composition of aromatic fuel. It has been deducted that higher levels of formed aromatic hydrocarbons had a high percentage of aromatic single-ring hydrocarbons when metals were loaded onto the Y-zeolite catalyst. López et al.^29^ utilized red clay and zeolite waste plastic pyrolysis. In pyrolysis, red clay was found to require higher temperatures than zeolite to achieve a catalytic effect. The products obtained at 440 °C in the case of using red clay as a catalyst can be compared with those obtained without using a catalyst at 500 °C, also, the quantity of oil and gases produced are larger besides the percentage of the aromatic substances in the produced liquids. Palomar et al., and Sivagami et al.^30, 31^ examined the influence of different types of zeolites on the quantity and quality of oil produced from catalytic pyrolysis of plastic. It can be concluded that the pyrolysis oils produced by catalytic pyrolysis include shorter chain molecules than the oils produced by thermal pyrolysis of waste plastic and can be utilized as an addition to conventional fuels in internal combustion engines.

Adrados et al.^32^ investigated the influence of cracking a plastic mixture with red mud as a catalyst. As a result, more light gases, more aromatic oil, and low waxy oil are produced. Also, red mud enhances the cracking and the aromatization processes. Additionally, it alters the liquids' chemical makeup, encouraging the synthesis of toluene and ethylbenzene at the expense of styrene. The FCC catalyst's influence, reaction temperature, and the ratio of catalyst to plastic were studied by Aisien et al.^33^, and it is reported that a ratio of 0.1 of catalyst to plastic reduced the amount of liquid oil and the generated char, yet there was an increase in the byproduct gases. The hydrocarbons in the liquid fractions ranged widely, mostly falling between C_4_ and C_17_. Abbas et al.^34^ studied the pyrolysis of PP using an equilibrium FCC catalyst to produce "fuel-like" hydrocarbons, where the effect of catalyst/polymer ratio, carrier gas type, and degradation temperature were also investigated. It has been determined that the degraded temperature of 450 °C and a ratio of 10% a catalyst/polymer produced the maximum yield of condensed products from the pyrolysis of PP. Using hydrogen as a reactive carrier gas increased the yield of condensed and paraffinic products.

From this review of the literature, it can be concluded that there are three different types of catalysts that can be employed in the cracking of plastics: silica-alumina, zeolite, and FCC catalysts, and the oils produced by using these catalysts in the pyrolysis process had characteristics similar to those of conventional fuel oils. Although these catalysts work effectively, their application is not practical because of the high manufacturing costs and significant process sensitivity to catalyst prices^35^. Therefore, the effect of a low-cost catalyst was studied in the pyrolysis process of waste plastic^36^. Nalluri et al.^37^ used fly ash as a low-cost catalyst in the pyrolysis of polyethylene using different mass fractions of the catalyst in the amount of 5%, 10% and 15%. It was found that the maximum oil yield was obtained at 5% and the production decreases by adding more catalyst. Ghodke^38^ used 10 wt% of CAT-1 as locally low-cost catalyst in the catalytic pyrolysis of municipal mixed plastic waste (MMPW). The maximum fuel yield of liquid fuel obtained using MMPW was 74.8 wt%. It is found that the produced fuel fractions have a wide range of carbon atom numbers between C_9_-C_18_. Luo et al.^39^ investigated the effect of Kaolin on the cracking of Polyethylene. It can be concluded that aliphatics and aromatics compose the majority of liquid oil, which typically have a carbon number ranges between C_6_ and C_20_. Also, Hakeem et al.^40^ used an Ahoko Kaolin catalyst with a 3.8 silica-to-alumina ratio in the pyrolysis of WPP. It is reported that using a Kaolin catalyst produced oil liquid with characteristics similar to traditional fuels. The recent research related to using low-cost natural catalysts for catalytic pyrolysis of polypropylene are summarized in Table 1^40–43^, and it can be concluded that using Kaolin results in the highest yield of oil with respect to other catalysts, equaling to 79.85 wt%.

**Table 1:** Recent research related to using low-cost catalysts for catalytic pyrolysis of polypropylene.

Plastic type	Catalyst	Result	References
Polypropylene (PP)	Alumina	- Concluded that using a 10% catalyst at pyrolysis temperature 465 °C produced 25 g of oil from catalytic pyrolysis of polypropylene - Using 25% of catalyst might be produce higher grade fuel for running automobile engines	^41^
Polypropylene (PP)	Kaolin	- The highest yield was produced with a catalyst-to-plastic ratio of 1:3, with a yield of 79.85 wt% - The liquid product comprises of hydrocarbons with various functional groups	^40^
Polypropylene (PP) and high-density polyethylene (HDPE)	Natural zeolite	- PP can be catalytically transformed to create liquid oil with a yield of up to 69.69 wt% - In the case of PP, the catalytically generated liquids hydrocarbons were C_5_–C_12_ (74.16%), C_13_–C_16_ (3.52%), and C_17_–C_20_ (22.32%)	^42^
Polypropylene (PP), and low-density polyethylene (LDPE)	N-clay	- The highest yield was 70.34 wt% - The oils derived from pyrolysis of PP and LDPE component like gasoline/kerosene	^43^

The objective of this research is to determine a low-cost Egyptian natural catalyst that can be used in the catalytic pyrolysis of waste polypropylene (WPP). Three different types of catalysts are examined, where these catalysts are collected from three different regions in Egypt; Kaolin from Aswan city, Hematite from Alwahat city, and white sand from Sinai city. The Kaolin is selected as a reference of comparison for the other catalysts, i.e. Hematite and white sand, since it is well known as an effective catalyst for the pyrolysis of WPP. Different ratios of catalysts to plastic were applied, such as 1:1, 1:2, 1:4, 1:6, and 1:8. The various catalysts utilized were elementally analyzed using the XRF analysis and the surface area was analyzed by the BET multi-point method. The thermogravimetric analysis is used to study the thermal degradation behavior of the WPP. The produced oil is characterized using the gas chromatography-mass spectrometry (GC–MS) and the differential scanning calorimetry (DSC).

## Experimental process

### WPP and catalyst preparation

Waste polypropylene (WPP) was utilized in the catalytic pyrolysis process, and it was obtained from Henkel Company for waste Plastic Collecting in Egypt^16, 44^. The WPP was cleaned, dried, and then crushed into smaller pieces of 3–5 mm for the thermal gravimetric test and the pyrolysis experiments. Three types of low-cost catalysts were examined; Kaolin, Hematite, and white sand, which were collected from Aswan, Alwahat, and Sinai cities in Egypt, respectively. All catalysts were ground to a fine powder using a ball mill for 5 h, so that the grain size of the final powder becomes less than 100 nm, and then were heated in a muffle for three hours at 500 °C for thermal activation. Afterwards, the different catalysts were characterized using the XRF analysis.

### Experimental procedure

The pyrolysis process test was conducted in a 1.5 kW electric furnace that served as the exterior heater for the vertical tube reactor, with a stainless-steel tube that has a 4 cm inner diameter and a length of 30 cm. The heating rate is controlled using a PID controller with K-type thermocouples to measure the reactor temperature.

**Figure 1:**
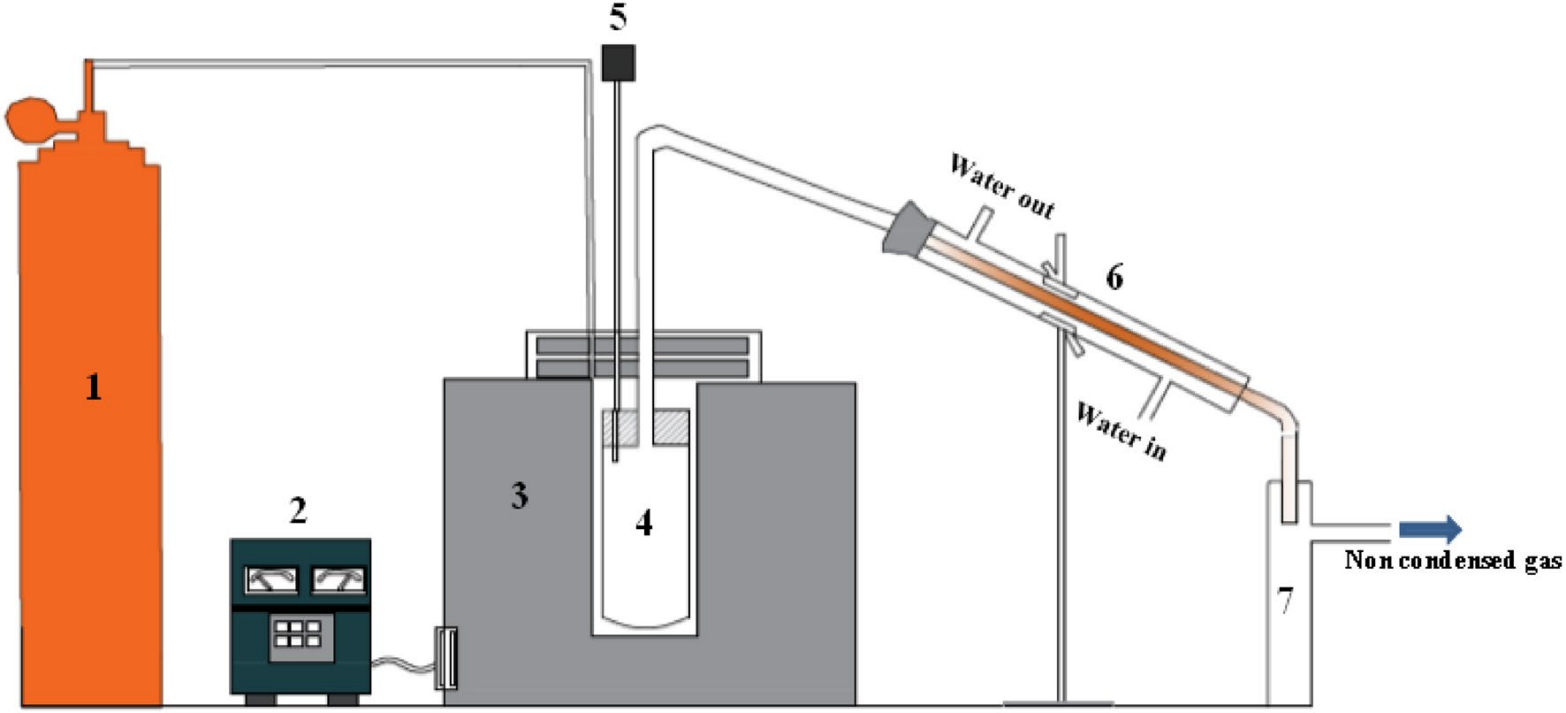
The pyrolysis system's schematic diagram, where (1) nitrogen bottle, (2) PID controller, (3) electrical heater, (4) pyrolysis reactor, (5) thermocouple, (6) condensing unit, and (7) oil collector.

The condensing unit was connected to the reactor exit, and nitrogen is pumped through the reactor with a flow rate of 80 mL/min during the pyrolysis process as illustrated in Fig. 1. The WPP and catalyst are added to the pyrolysis reactor, then the reactor is heated to 500 °C at a rate of 5 °C/min under the flow of nitrogen. Afterwards, the reactor temperature is maintained constant for 30 min until the pyrolysis process is finished. The gaseous products are condensed at room temperature using a condenser that is attached to the reactor's outlet, and a gas bag is used to collect the uncondensed gases. Finally, the mass balance method is used to calculate the gas production after weighing the collected liquid and the char that has been deposited in the reactor^45^. The following equations were employed to calculate the mass proportion of oil, char, and non-condensable gases:

1 $$\mathrm{Percentage\,of\,oil\,yield},\mathrm{ Oil }(\mathrm{wt\%})=\frac{\mathrm{ Mass\,of\,Oil}}{\mathrm{Mass\,of\,WPP}}\times 100$$

2 $$\mathrm{Percentage\,of\,char\,yield},\mathrm{ Char }\,\,\left(\mathrm{wt\% }\right)=\frac{\mathrm{ Mass\,of\,Char}}{\mathrm{Mass\,of\,WPP}} \times 100$$

3 $${\text{Percentage of gas yield}},{\text{ Gas}}\,\,\left( {{\text{wt}}.\% } \right) \, = { 1}00 \, - \, \left( {{\text{Oil}}\% \, + {\text{ Char}}\% } \right)$$

### Characterization

#### Characterization of WPP

**Figure 2:**
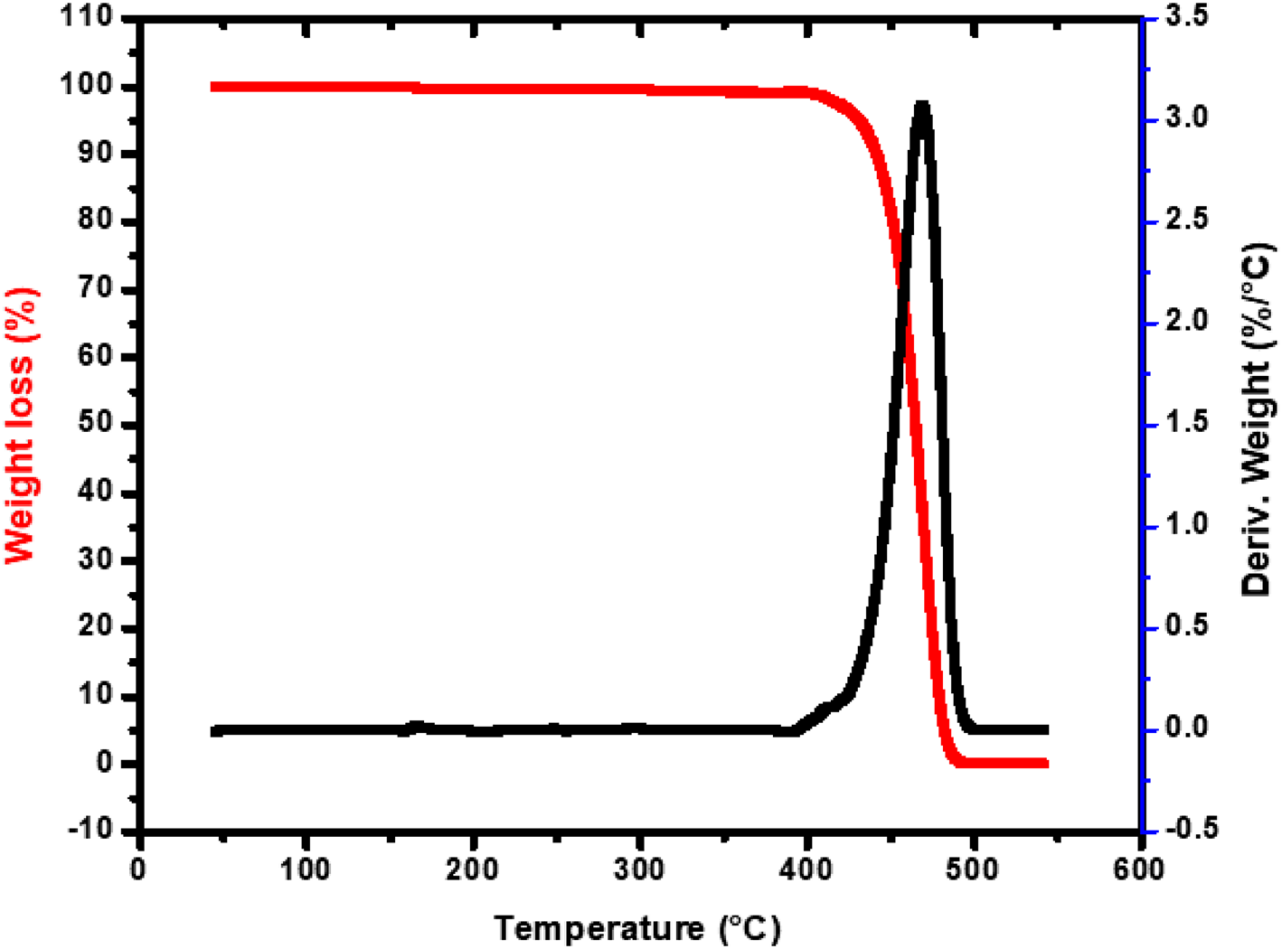
Temperature gradient (TG) and differential thermal (DT) curves for WPP.

Temperature gradient (TG) and differential thermal (DT) are analytical measurements used to examine the thermal degradation behavior of WPP using an SDT Q600 USA thermogravimetric analyzer. WPP was heated in a nitrogen environment at a rate of 10 °C/min from room temperature to 1000 °C. The TG and DT curves of WPP using nitrogen gas and heating the WPP at a rate of 10 °C/min are shown in Fig. 2. It can be shown that the greatest weight loss of WPP due to thermal degradation occurred at 465 °C. The thermal cracking of WPP begins at a temperature of 410 °C and at 468 °C reaches its maximum rate. At 485 °C, the WPP is completely converted into gases^46^, which can be seen in the DT curve in Fig. 2.

#### Characterization of catalysts

The technical composition of Kaolin, Hematite, and white sand were identified by X-Ray Fluorescence (XRF), and the outcomes are shown in Table 2. The identified elements were supplied as a percentage of the elements in the overall sample that are represented as oxides. It was observed that the majority of the compounds in the Kaolin sample are silicon oxide (SiO_2_) and aluminum oxide (Al_2_O_3_), whereas the majority of the compounds in the Hematite and white sand samples are ferric oxide (Fe_2_O_3_) and silicon oxide, respectively; however, trace levels of many other oxides were noticed. The specific surface area of the three examined catalysts were determined by the BET multi-point method using the 3H-2000PSI system, and the results are presented in Table 3. It can be observed from Table 3 that the specific surface area of kaolin, 51.66 m^2^/g, is nearly double the specific surface area of Hematite, which is 29.62 m^2^/g, and also greater than white sand. The pore volume per unit mass is also presented in Table 3, and it can be deduced that it is directly proportional to the specific surface area, since they are coupled, which assures the accuracy of measurements. Based on the specific area, Kaolin's high surface area of reaction per unit mass indicates that it is the most effective catalyst for use in catalytic pyrolysis of WPP among the investigated catalysts.

**Table 2:** Percentage of chemical composition of the different catalysts used in the pyrolysis of WPP.

Oxides %	Catalyst		
	Kaolin	Hematite	White sand
SiO_2_	**60.06**	6.23	**97.21**
Al_2_O_3_	**29.52**	0.66	0.89
Fe_2_O_3_	5.33	**88.37**	0.48
CaO	0.33	0.84	0.1
MgO	0.33	0.41	0.01
SO_3_	0.47	1.47	0.15
K2O	0.39	0.24	0.07
Na_2_O	0.15	0.2	0.15
TiO_2_	2.08	0.17	0.15
Cl	0.01	0.04	0.01
LOI	1.22	1.71	0.69

Significant values are given in bold.

**Table 3:** Surface area analysis of the different catalysts used in the pyrolysis of WPP.

Samples	S_BET_ (m^2^/g)	Pore volume per unit mass (cc/g)	Pore size (nm)
Kaolin	**51.66**	0.441	9.51
Hematite	**29.62**	0.197	28.46
White sand	37.82	0.217	20.29

Significant values are given in bold.

#### Characterization of oil

The oil produced by the pyrolysis process was qualitatively estimated using the GC–MS. GC system 7010B GC/TQ inert mass spectrometry with triple Axis had been used for the analysis of matched chromatogram peaks. The carrier gas utilized was Helium. The Wiley Registry/NIST Library was used to identify peaks with the highest probability and quality of greater than 80%. Differential scanning calorimetry (DSC) analysis was used to study the thermal behavior of the produced oil. The oil was placed in an aluminum crimp cell and heated at a heating rate of 10 °C/min from room temperature to 400 °C in a nitrogen atmosphere using SETARAM instrumentation, Themys one plus model, and the peak transition onset temperatures were recorded^47^.

## Experimental results

### Yield of products

#### Yield of Products at different ratios of Kaolin catalyst to WPP

**Figure 3:**
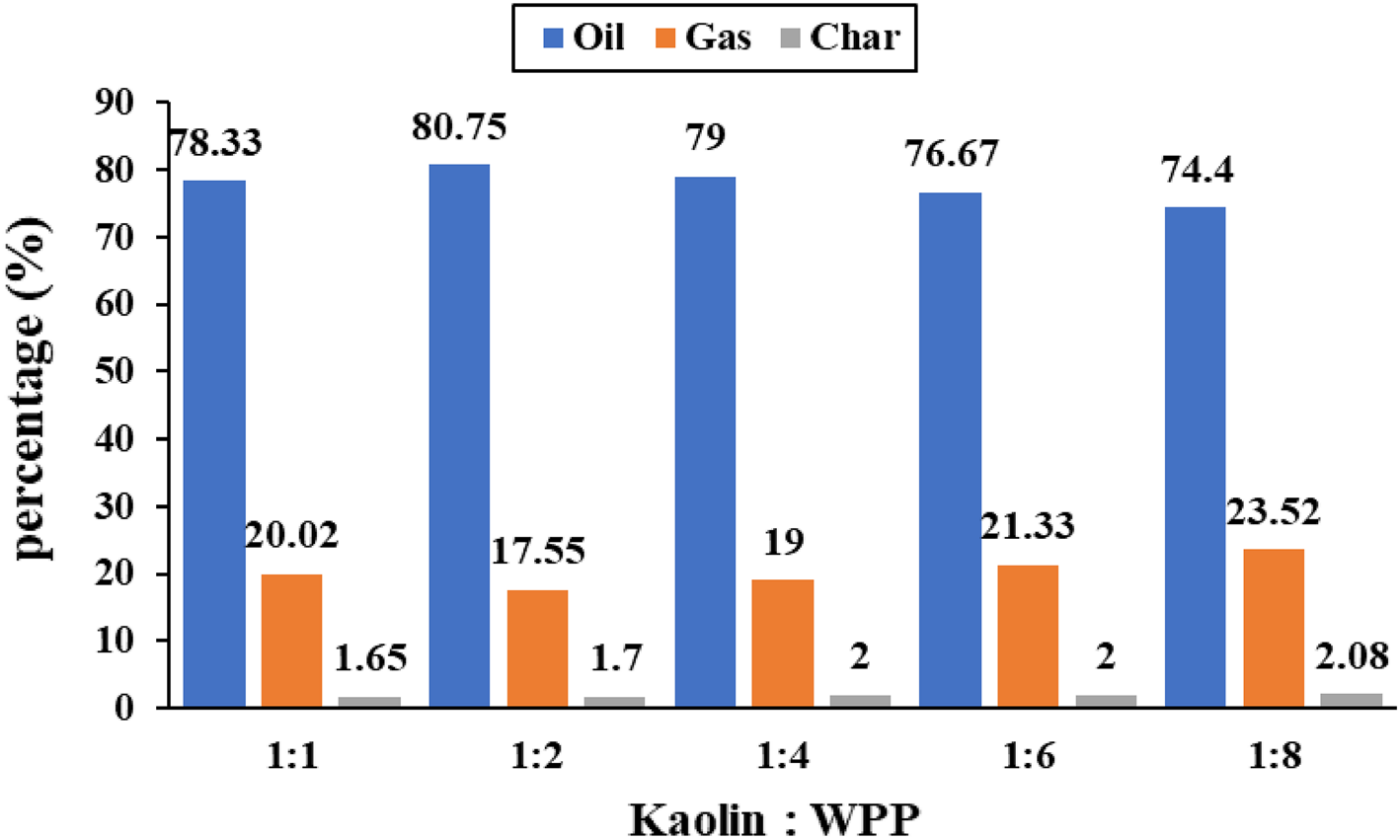
Product’s yield (wt%) of the pyrolysis of WPP at various ratios of Kaolin catalyst to WPP.

Product’s yield due to pyrolysis of WPP at various ratios of Kaolin catalyst to WPP are stated in Fig. 3. As the Kaolin to WPP ratio increases from 1:8 to 1:2, the liquid yield increased from 74.4 to 80.75 wt%, while the gas yield decreased from 23.52 to 17.55 wt%. The Kaolin to WPP ratio of 1:2 resulted in the highest yield of liquid oil, i.e., 80.75 wt%. The oil yield was reduced to 78.33 wt% as a result of the catalyst to plastic ratio being increased further to 1:1. This indicates increasing or decreasing the Kaolin to WPP ratio from 1:2 will decrease the oil yield and increase the gas yield, i.e., Kaolin to WPP ratio of 1:2 is the optimum for oil yield.

#### Yield of products at different ratios of Hematite catalyst to WPP

**Figure 4:**
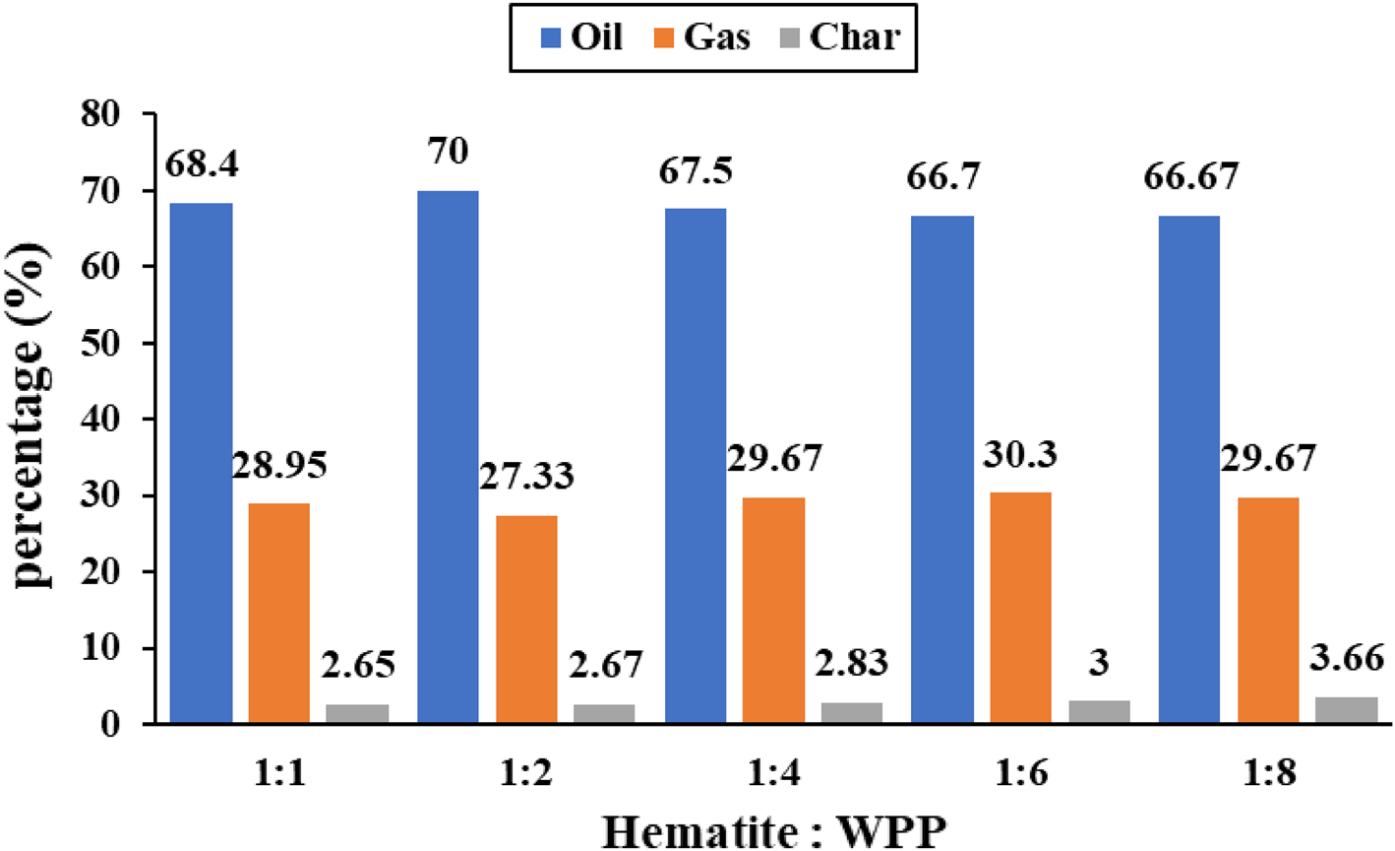
Product’s yield (wt%) due to pyrolysis of WPP at various ratios of Hematite catalyst to WPP.

The product yield due to the catalytic pyrolysis of WPP with different ratios of Hematite catalyst to plastic are shown in Fig. 4. It can be observed that increasing the ratio of Hematite to WPP from 1:8 to 1:2 leads to an increase in the oil yield, such that it has increased from 66.67 wt% to 70 wt%. Then the oil yield was reduced to 68.4 wt% because of increasing this ratio to 1:1. It can be concluded that using Hematite in the catalytic pyrolysis of WPP has a weak effect in increasing the oil yield. This is consistent with Cybris and Soudan^48^ conclusion that iron oxide has less effect at a lower heating rate and temperature up to 600 °C.

#### Yield of Products at different ratios of white sand catalyst to WPP

**Figure 5:**
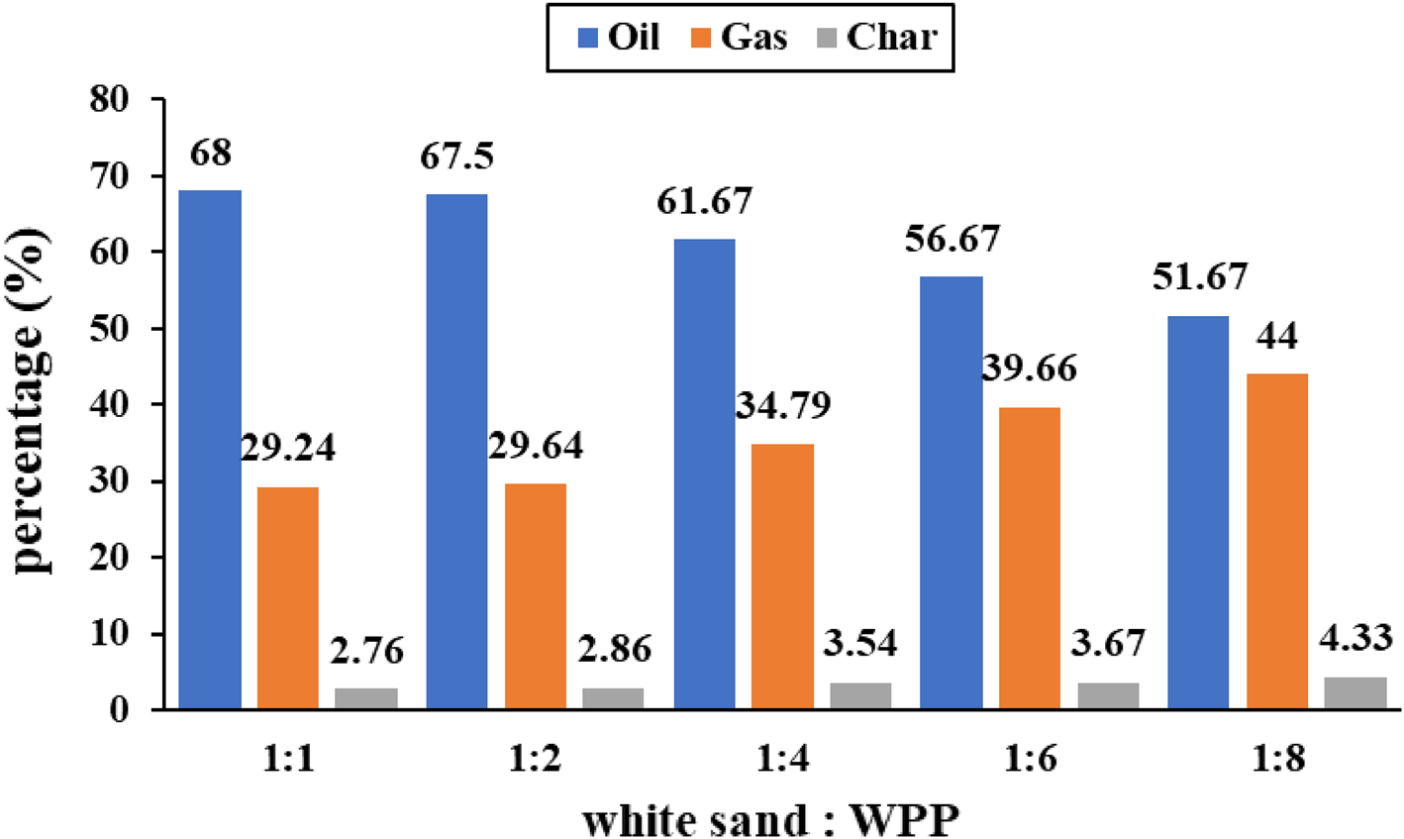
Product’s yield (wt%) due to pyrolysis of WPP at various ratios of white sand catalyst to WPP.

The product yield due to catalytic pyrolysis of WPP at various ratios of White sand catalyst to WPP are displayed in Fig. 5. It has been found that increasing the ratio of white sand to WPP from 1:8 to 1:1, caused an increase in the liquid yield from 51.67 to 68 wt%, whereas the gas yield decreased from 44 to 29.24 wt%. The highest yield of produced gases, i.e., 44 wt% has been obtained by catalytic pyrolysis of WPP using a White sand to WPP ratio of 1:8. The oil yield due to non-catalytic pyrolysis of WPP and the maximum oil yield due to using different types of catalysts are displayed in Table 4. The yield of oil due to non-catalytic pyrolysis of WPP was 70 wt% of liquid oil, 24.3 wt% of gas and 5.7 wt% of solid char. The highest liquid yield due to using Kaolin, Hematite and white sand are 80.75 wt%, 70 wt%, and 68 wt%, respectively. The liquid yield due to catalytic pyrolysis of WPP increased when Kaolin is used but decreased when white sand is used. Using Kaolin as a catalyst resulted in the highest yield of liquid oil. The rise in liquid yield due to using Kaolin as a catalyst represents a significant improvement in the thermal cracking of WPP, and that is due to Kaolin’s acidity, mesoporous surface area and, high Si/Al ratio^40^.

**Table 4:** The catalyst to WPP ratio has resulted in the highest oil yield (wt%) due to catalytic and non-catalytic pyrolysis of WPP.

Catalyst	Yield (wt%)			
	Catalyst:WPP	Oil	Gas	Char
No catalyst	–	70	24.3	**5.7**
Kaolin	1:2	**80.75**	17.55	1.7
Hematite	1:2	70	27.33	2.67
White sand	1:1	68	**29.24**	2.76

Significant values are given in bold.

Using just Si as a catalyst, which is almost the case of white sand, is not sufficient to improve the oil yield, since the oil yield in case of no catalyst is higher than in case of using white sand as a catalyst. However, adding Al_2_O_3_ to Silica, which is the case of using Kaolin as a catalyst, has improved the oil yield a lot compared to the no catalyst case as well as the other catalysts, i.e., white sand and Hematite catalysts, as can be seen in Table 4. Therefore, it is highly recommended to study the influence of Al_2_O_3_ to Silica ratio on the oil yield and quality during the catalytic pyrolysis of WPP, in order to determine the optimum ratio. Using white sand as a catalyst has resulted in decreasing the yield of oil but increasing the gaseous compound^49^. So, it can be concluded that if the objective of the pyrolysis process is liquid fuel, then it is preferred to use Kaolin as a catalyst, while if the objective is gaseous fuel, then it is preferred to use white sand.

### Quality of oil

**Figure 6:**
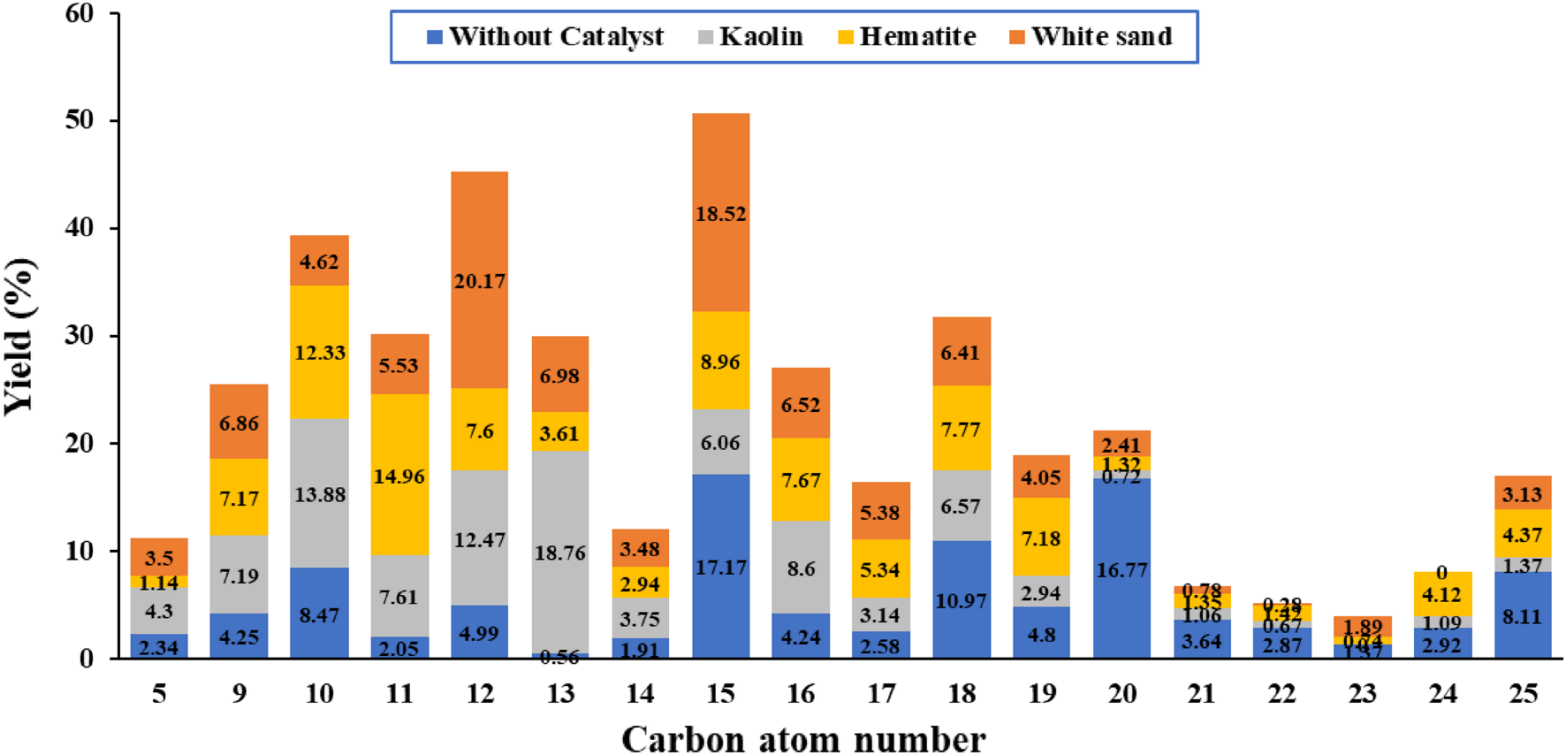
Carbon atom number of produced oil from non-catalytic and catalytic pyrolysis of WPP.

The highest yield of oil from the catalytic pyrolysis of WPP has been analyzed using the GC–MS analyzer, and the results are presented and discussed in this section. The catalyst to WPP ratio that has resulted in the highest oil yield, as a function of catalyst type, has been previously observed in Table 4. The number of carbon atoms of the produced oil from the catalytic and non-catalytic pyrolysis of WPP is presented in Fig. 6 and Table 5. It can be concluded from Table 5 that the produced oil in case of non-catalytic pyrolysis of WPP had a big percentage of heavy oils, i.e., > C_15_. However, using a catalyst during the pyrolysis of WPP has decreased the percentage of heavy oils, and increased the percentage of light oil, i.e., C_5_–C_10_, as well as the average weight oil, C_11_–C_15_^50^, as can be seen in Table 5.

**Table 5:** The weight percentage of the produced oil according to the Carbon atom number as a function of the type of catalyst, based on the GC–MS analysis.

Catalyst	Carbon number range		
	Weight % of		
	C_5_–C_10_	C_11_–C_15_	> C_15_
No catalyst	**15.06** (Lowest)	26.68	**58.26** (Highest)
Kaolin	**25.37** (Highest)	48.65	**25.98** (Lowest)
Hematite	20.64	38.07	41.9
White sand	19.98	49.68	30.34

Significant values are given in bold.

**Figure 7:**
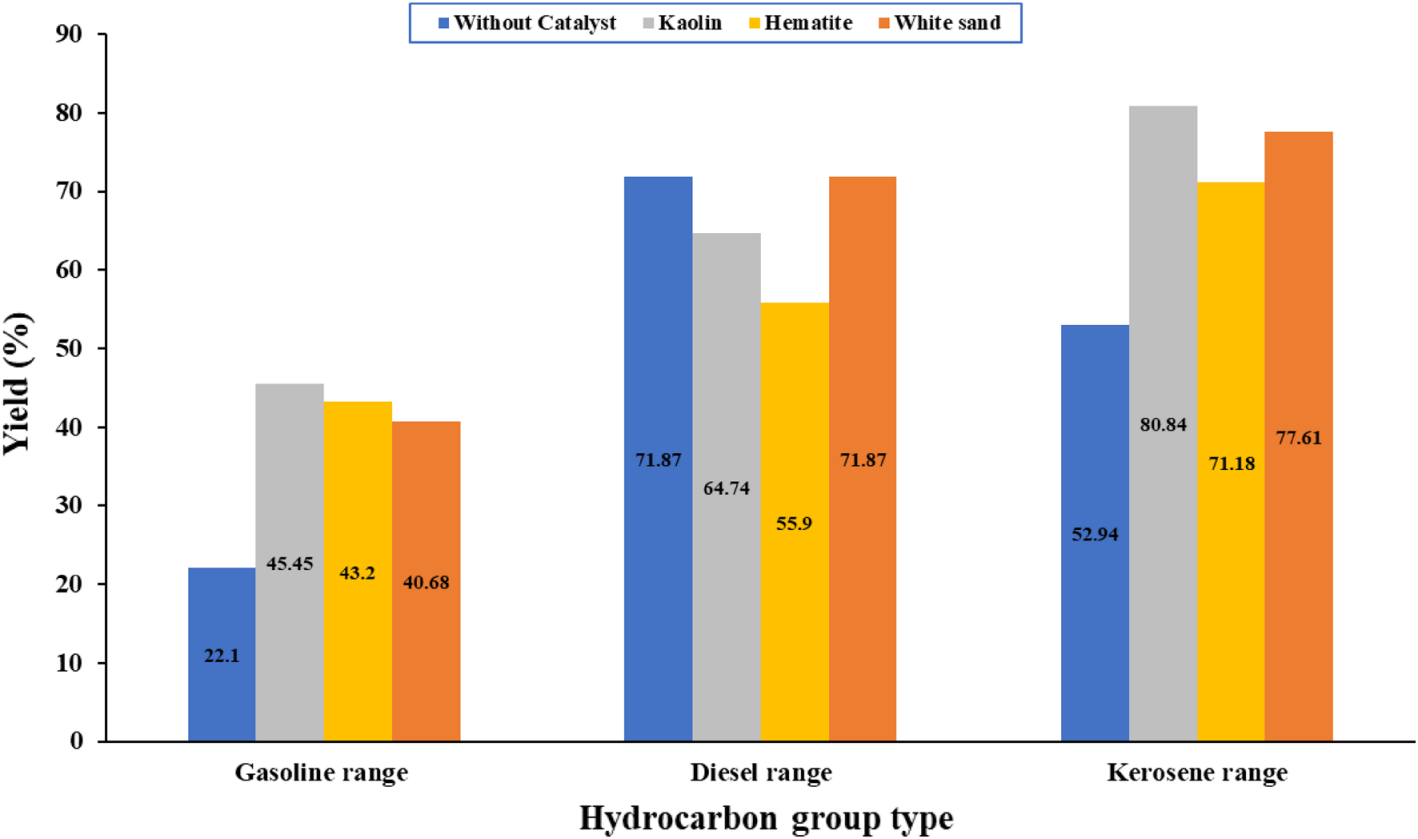
Weight percentage of the produced oil from the non-catalytic and catalytic pyrolysis of WPP as a function of the fuel group, i.e., gasoline (C_4_–C_12_), Kerosene (C_10_–C_18_) and diesel (C_12_–C_23_).

Using Kaolin in the catalytic pyrolysis of WPP produced oil with the lowest percentage of heavy oil, i.e., 25.98%, and the highest percentage of light oil, i.e., 25.37%, as compared to other catalysts, i.e., Hematite and white sand. Therefore, it can be concluded from Table 5 that if the objective of the pyrolysis process is to produce light weight hydrocarbons, then it is better to use Kaolin catalyst than Hematite and white sand. The weight percentage of produced oil from the non-catalytic and catalytic pyrolysis of WPP has been classified according to the fuel group, i.e., gasoline (C_4_–C_12_), kerosene (C_10_–C_18_) and diesel (C_12_–C_23_)^51^, and the results are presented in Fig. 7. Therefore, it can be concluded from Fig. 7 that the Kaolin catalyst has increased the percentage of gasoline and Kerosene in the produced oil in comparison to other catalysts, i.e., Hematite, white sand, and the non-catalyst case, which promotes the Kaolin catalyst to be used for the pyrolysis of WPP if the objective is light oils like gasoline and kerosene.

**Figure 8:**
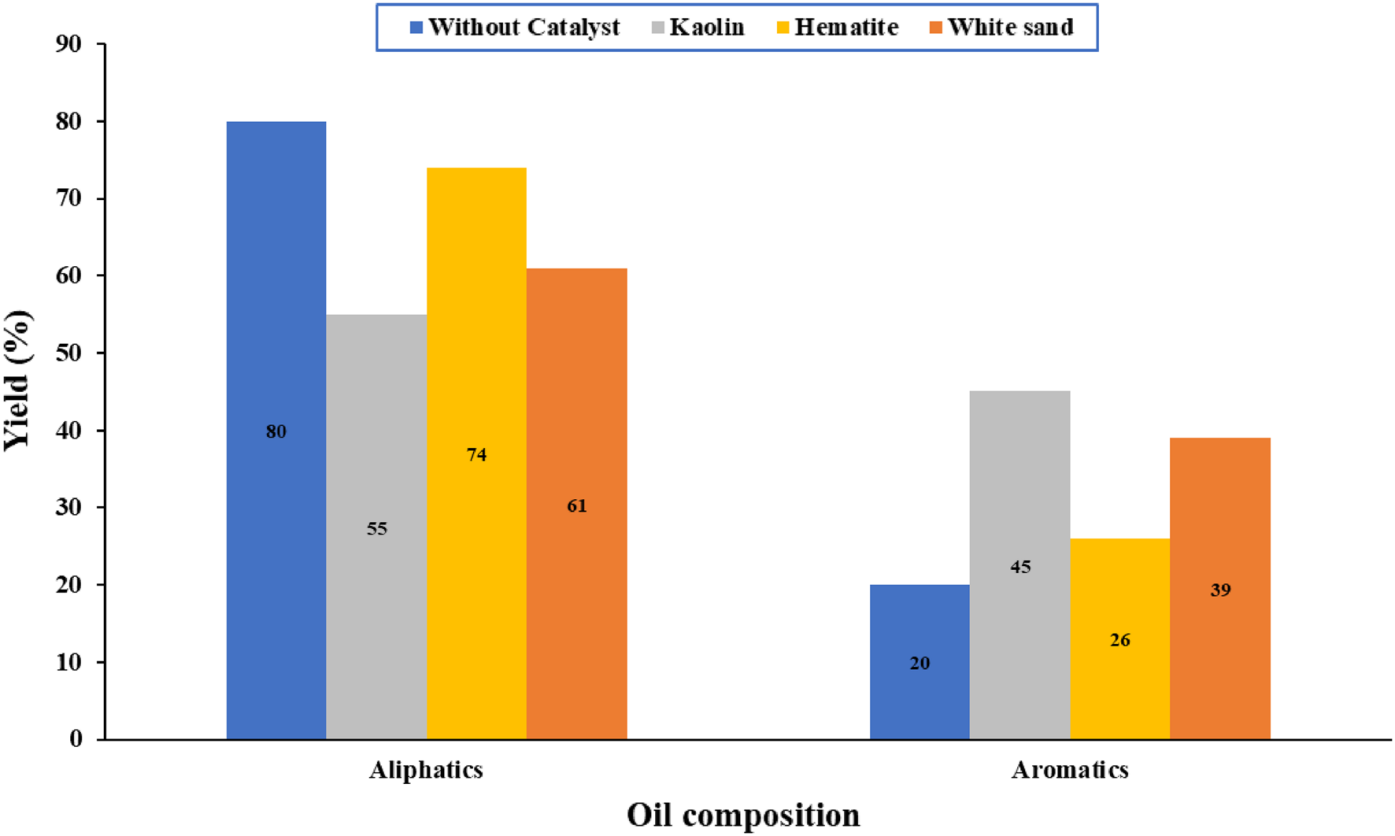
Oil composition of non-catalytic and catalytic pyrolysis of WPP.

**Figure 9:**
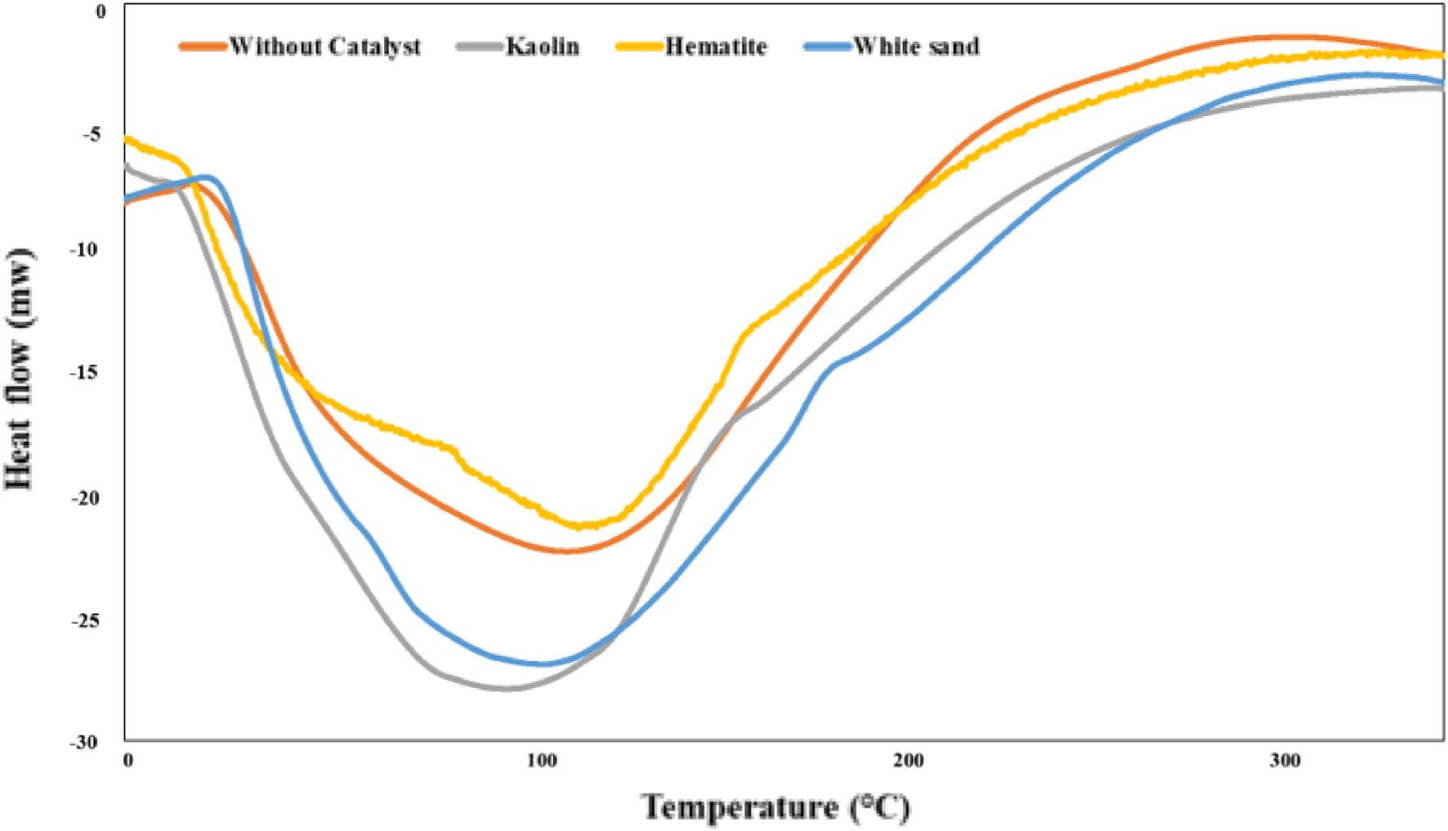
DSC curves of Oil produced from non-catalytic and catalytic pyrolysis of WPP.

The produced oil from the non-catalytic and catalytic pyrolysis of WPP is a combination of hydrocarbons having carbon atoms ranging from C_5_ to C_25_. The composition of the produced oil because of WPP's non-catalytic and catalytic pyrolysis in terms of the aliphatic (alkanes, alkenes, cycloalkanes) and aromatics groups are presented in Fig. 8^52^. It can be concluded from Fig. 8 that adding Kaolin to the WPP during the pyrolysis process resulted in the production of oil with the highest percentage of aromatic oil, i.e., 45%, as compared to other catalysts, i.e., Hematite and white sand, and the percentage of aliphatic oil was highest, i.e., 80%, in case of non-catalytic pyrolysis. As a result, adding Kaolin to the WPP during the pyrolysis process enhances the oil's quality, i.e., it produces lighter aromatic oil with a high percentage of gasoline range. Differential scanning calorimetry (DSC) analysis was used to study the thermal behavior of the highest yield of oil produced from the catalytic pyrolysis of WPP as shown in Fig. 9, and the results are summarized in Table 6. It can be observed that all samples have a typical endothermic peak, and the boiling points of the studied oil samples in this research range from 82 to 115 °C. The oil produced from the catalytic pyrolysis of WPP using Kaolin catalyst had the lowest boiling point, i.e. 82 °C, while the oil produced from the thermal cracking of WPP without a catalyst had the highest boiling point, which is 115 °C. Therefore, it can be concluded that the oil produced by using the Kaolin catalyst is lighter than other oils which is in line with the oil composition presented in Fig. 8. The percentage of the aromatics are highest and the percentage of the aliphatics are lowest in the oil produced by using the Kaolin catalyst compared to other produced oils. It can also be concluded that the oil produced using the Kaolin catalyst can be easily used in a diesel engine without affecting its performance due to its low boiling temperature.

**Table 6:** Boiling point of produced oil.

Oil sample	Boiling point (°C)
Without catalyst	115
Kaolin	82
Hematite	104
White sand	95

### Feasibility of catalyst

The cost of oil production due to the different catalysts used is compared and the results are presented in Table 7. The comparison is based on a 0.5 kg catalyst used in the pyrolysis of WPP, and the ratio of the catalyst to WPP that has resulted in the highest oil yield, this was compiled in Table 4. The Kaolin catalyst has the lowest cost of oil production, as can be observed, i.e., 0.28 $/kg of oil, while the Hematite catalyst has the highest production cost, which is 3.57 $/kg of oil. Therefore, it can be concluded that Kaolin is more economical than Hematite and white sand in the pyrolysis of WPP, and it is recommended to be taken as a feasible catalyst for the pyrolysis of WPP. Also, despite the fact that Kaolin is the cheapest catalyst it improves the quantity as well as the quality of the formed oil in comparison to Hematite, white sand, and the non-catalytic case.

**Table 7:** Price of catalyst per kg of produced oil.

Catalyst	Catalyst mass [kg]	*Catalyst:WPP [–]	**Catalyst price [$/kg]	Oil yield [kg]	Catalyst price/kg of oil [$/kg]
Kaolin	0.5	1:2	0.45	0.8	0.28
Hematite	0.5	1:2	5	0.7	3.57
White sand	0.5	1:1	0.5	0.68	0.37

*The presented ratios are the ratios that have resulted in the highest oil yield.

**The given prices are based on^53^.

## Conclusions

The objective of this research is to determine a low-cost Egyptian natural catalyst that can be used in the catalytic pyrolysis of waste polypropylene (WPP). Three different types of catalysts are examined, where these catalysts are collected from three different regions in Egypt; Kaolin from Aswan city, Hematite from Alwahat city and white sand from Sinai city. Different ratios of catalysts to plastic were applied, such as 1:1, 1:2, 1:4, 1:6, and 1:8. It can be concluded from the performed research that;

1. Thermal cracking without a catalyst produced a yield of 70 wt% of liquid oil, 24.3 wt% gas and 5.7 wt% char.
2. The maximum oil yield in the case of using Hematite and white sand as a catalyst were 70 wt% and 68 wt%, respectively.
3. The ratio of 1:2 of the Kaolin to the plastic resulted in the highest oil yield, i.e. 80.75 wt%, and it has the lowest percentage of heavy oil, which is 25.98%, and the highest percentage of light oil, i.e. 25.37%.
4. The ratio of 1:8 of the white sand to the plastic produced the highest gas yield, which is 44 wt%.
5. It is preferred to use Kaolin as a catalyst, if the objective of the pyrolysis process is liquid fuel, while if the objective is a gaseous fuel, then it is preferred to use white sand.
6. Kaolin has the lowest cost of oil production compared to Hematite and white sand, which is 0.28 $/kg of oil, and it can be concluded that Kaolin is an economical catalyst that improves the quality, as well as the quantity of the produced oil in comparison to Hematite, white sand, and the non-catalytic case.

## Data Availability

Information sharing and material requests should be emailed to A.I. Eldahshory.

## References

[1] Qi P, Chang G, Wang H, Zhang X, Guo Q (2018). Production of aromatic hydrocarbons by catalytic co-pyrolysis of microalgae and polypropylene using HZSM-5. J. Anal. Appl. Pyrol..

[2] Peng Y, Wang Y, Ke L, Dai L, Wu Q, Cobb K, Ruan R (2022). A review on catalytic pyrolysis of plastic wastes to high-value products. Energy Convers. Manage..

[3] Capuano, L. International energy outlook 2018 (IEO2018). *US Energy Information Administration (EIA)*: Washington, DC, USA, 2018, 21 (2018)‏.

[4] Abdel-Shafy HI, Mansour MS (2018). Solid waste issue: Sources, composition, disposal, recycling, and valorization. Egypt. J. Pet..

[5] Miteva K, Aleksovski S, Bogoeva-Gaceva G (2016). Catalytic pyrolysis of waste plastic into liquid fuel. Zaštita Materijala.

[6] Emara K (2023). Sustainable solid waste management in rural areas: A case study of Fayoum governorate, Egypt. Energy Nexus.

[7] Gomaa RK, Atef A, Mostafa A (2022). Use of environment friendly recycled building materials in Egypt. J. Al-Azhar Univ. Eng. Sector.

[8] Abdellatif, G., Mahmoud, A.S., Peters, R.W., & Mostafa, M.K. Waste plastics and microplastics in Africa: Negative impacts and opportunities. In* 2021 AIChE Annual Meeting. New York,* NY, USA: AIChE (2021).

[9] Kyaw KT, Hmwe CSS (2015). Effect of various catalysts on fuel oil pyrolysis process of mixed plastic wastes. Int. J. Adv. Eng. Technol..

[10] Maddah HA (2016). Polypropylene as a promising plastic: A review. Am. J. Polym. Sci.

[11] Lambert S, Wagner M (2017). Environmental performance of bio-based and biodegradable plastics: The road ahead. Chem. Soc. Rev..

[12] Wojnowska-Baryła I, Bernat K, Zaborowska M (2022). Plastic waste degradation in landfill conditions: The problem with microplastics, and their direct and indirect environmental effects. Int. J. Environ. Res. Public Health.

[13] de Paula FG, de Castro MC, Ortega PF, Blanco C, Lavall RL, Santamaría R (2018). High value activated carbons from waste polystyrene foams. Microp. Mesoporous Mater..

[14] Wu SL, Kuo JH, Wey MY (2019). Thermal degradation of waste plastics in a two-stage pyrolysis-catalysis reactor over core-shell type catalyst. J. Anal. Appl. Pyrol..

[15] Ragaert K, Delva L, Van Geem K (2017). Mechanical and chemical recycling of solid plastic waste. Waste Manage..

[16] Eldahshory, A.I., Emara, K., Abd-Elhady, M.S., & Ismail, M.A. High quality and maximizing the production of CNTs from the pyrolysis of waste polypropylene. *Arab. J. Sci. Eng.* 1–12 (2022).‏

[17] Mishra R, Kumar A, Singh E, Kumar S (2023). Recent research advancements in catalytic pyrolysis of plastic waste. ACS Sustain. Chem. Eng..

[18] Aguado J, Serrano DP, San Miguel G, Castro MC, Madrid S (2007). Feedstock recycling of polyethylene in a two-step thermo-catalytic reaction system. J. Anal. Appl. Pyrol..

[19] Moustakas, K., & Loizidou, M. Solid waste management through the application of thermal methods. *Waste Manage* (2010).

[20] Sharuddin, S.D.A., Abnisa, F., Daud, W.M.A.W., & Aroua, M.K. Pyrolysis of plastic waste for liquid fuel production as prospective energy resource. *In IOP Conference Series: Materials Science and Engineering* (Vol. 334, p. 012001). IOP Publishing (2018).‏

[21] Christopher FJ, Kumar PS, Jayaraman L, Rangasamy G (2023). Assessment of product distribution of plastic waste from catalytic pyrolysis process. Fuel.

[22] Kunwar B, Cheng HN, Chandrashekaran SR, Sharma BK (2016). Plastics to fuel: A review. Renew. Sustain. Energy Rev..

[23] Papuga S, Djurdjevic M, Ciccioli A, Vecchio Ciprioti S (2022). Catalytic pyrolysis of plastic waste and molecular symmetry effects: A review. Symmetry.

[24] Susastriawan AAP, Sandria A (2020). Experimental study the influence of zeolite size on low-temperature pyrolysis of low-density polyethylene plastic waste. Thermal Sci. Eng. Progress.

[25] Onwudili JA, Muhammad C, Williams PT (2019). Influence of catalyst bed temperature and properties of zeolite catalysts on pyrolysis-catalysis of a simulated mixed plastics sample for the production of upgraded fuels and chemicals. J. Energy Inst..

[26] Linh NTT, Tuan PD (2020). Effects of using ZSM-5 and a rock-reforming mineral as catalysts on liquid fraction collected from polypropylene pyrolysis. Vietnam J. Chem..

[27] Wang J, Jiang J, Wang X, Wang R, Wang K, Pang S, Ragauskas AJ (2020). Converting polycarbonate and polystyrene plastic wastes intoaromatic hydrocarbons via catalytic fast co-pyrolysis. J. Hazard. Mater..

[28] Akubo K, Nahil MA, Williams PT (2019). Aromatic fuel oils produced from the pyrolysis-catalysis of polyethylene plastic with metal-impregnated zeolite catalysts. J. Energy Inst..

[29] López A, De Marco I, Caballero BM, Laresgoiti MF, Adrados A, Aranzabal A (2011). Catalytic pyrolysis of plastic wastes with two different types of catalysts: ZSM-5 zeolite and Red Mud. Appl. Catal. B.

[30] Palomar-Torres A, Torres-Jimenez E, Kegl B, Bombek G, Volmajer-Valh J, Lešnik L (2023). Catalytic pyrolysis of plastic wastes for liquid oils’ production using ZAP USY zeolite as a catalyst. Int. J. Environ. Sci. Technol..

[31] Sivagami K, Kumar KV, Tamizhdurai P, Govindarajan D, Kumar M, Nambi I (2022). Conversion of plastic waste into fuel oil using zeolite catalysts in a bench-scale pyrolysis reactor. RSC Adv..

[32] Adrados A, De Marco I, Caballero BM, López A, Laresgoiti MF, Torres A (2012). Pyrolysis of plastic packaging waste: A comparison of plastic residuals from material recovery facilities with simulated plastic waste. Waste Manage..

[33] Aisien ET, Otuya IC, Aisien FA (2021). Thermal and catalytic pyrolysis of waste polypropylene plastic using spent FCC catalyst. Environ. Technol. Innov..

[34] Abbas-Abadi MS, Haghighi MN, Yeganeh H, McDonald AG (2014). Evaluation of pyrolysis process parameters on polypropylene degradation products. J. Anal. Appl. Pyrol..

[35] Weibing, D., Liang, J., & Anderson, L.L. Hydrocracking of waste plastics to clean liquid fuels. *University of Utah, Department of Chemical and Fuels Engineering. Salt Lake City, UT*, **841**(12), 3290 (2014).‏

[36] Fadillah G, Fatimah I, Sahroni I, Musawwa MM, Mahlia TMI, Muraza O (2021). Recent progress in low-cost catalysts for pyrolysis of plastic waste to fuels. Catalysts.

[37] Nalluri P, Kumar PP, Sastry MC (2021). Experimental study on catalytic pyrolysis of plastic waste using low-cost catalyst. Mater. Today Proc..

[38] Ghodke PK (2021). High-quality hydrocarbon fuel production from municipal mixed plastic waste using a locally available low-cost catalyst. Fuel Commun..

[39] Luo W, Fan Z, Wan J, Hu Q, Dong H, Zhang X, Zhou Z (2021). Study on the reusability of kaolin as catalysts for catalytic pyrolysis of low-density polyethylene. Fuel.

[40] Hakeem IG, Aberuagba F, Musa U (2018). Catalytic pyrolysis of waste polypropylene using Ahoko kaolin from Nigeria. Appl. Petrochem. Res..

[41] Jha KK, Kannan TTM, Senthilvelan N (2021). Optimization of catalytic pyrolysis process for change of plastic waste into fuel. Mater. Today Proc..

[42] Liandi AR, Setyono MH, Aziz I, Siregar YDI (2023). Pyrolysis of PP and HDPE from plastic packaging waste into liquid hydrocarbons using natural zeolite Lampung as a catalyst. Case Stud. Chem. Environ. Eng..

[43] Adoga SO, Igbum OG, Okopi G, Ikyenge BA, Ochi OI, Ode P (2022). Catalytic pyrolysis of low density polyethylene and polypropylene wastes to fuel oils by N-clay. Ovidius Univ. Ann. Chem..

[44] Sweah J, Z., Mohammed, A.J., Malik, F.H. (2023). Preparation and study of mechanical properties of polymer blends from recycled materials polymethylmethacryalate with different ratios of polyester and shilajit. Egypt. J. Chem..

[45] Mabood F, Shah J, Jan MR (2010). Catalytic conversion of waste low density polyethylene into valuable products. J. Chem. Soc. Pak..

[46] Ng, H.M., Saidi, N.M., Omar, F.S., Ramesh, K., Ramesh, S., & Bashir, S. Thermogravimetric analysis of polymers*. Encyclop. Polym. Sci. Technol.* 1–29 (2002).

[47] Saudagar WS, Sidram GP, Baburo GS, Agarwal G, Agarwal S, Gadgeppa BO (2021). Development and characterization of terminalia arjuna phospholipid complex and its tablet formulation by Qbd approach. Int. J. Life Sci. Pharma Res.

[48] Cypres, R., & Soudan-Moinet, C. Pyrolysis of coal and iron oxides mixtures. 1. Influence of iron oxides on the pyrolysis of coal. *Fuel* **59**(1), 48–54 (1980).‏

[49] Murata K, Brebu M, Sakata Y (2010). The effect of silica–alumina catalysts on degradation of polyolefins by a continuous flow reactor. J. Anal. Appl. Pyrol..

[50] Ryu HW, Kim DH, Jae J, Lam SS, Park ED, Park YK (2020). Recent advances in catalytic co-pyrolysis of biomass and plastic waste for the production of petroleum-like hydrocarbons. Biores. Technol..

[51] Shumba, T. Catalytic pyrolysis of plastic waste to produce diesel-like fuel (*Doctoral Dissertation*) (2021).

[52] Jung SH, Cho MH, Kang BS, Kim JS (2010). Pyrolysis of a fraction of waste polypropylene and polyethylene for the recovery of BTX aromatics using a fluidized bed reactor. Fuel Process. Technol..

[53] [online] Retrieved May. 20, 2023 from URL: https://arabic.alibaba.com/p-detail/Metakaolin-1600311759296.htmlspm=a2700.galleryofferlist.normal_offer.d_image.4a1d5a4dsU2abf.

